# Translation, adaptation, and psychometric properties of the Brazilian-Portuguese version of the Quality of Life in Children with Vernal Keratoconjunctivitis questionnaire

**DOI:** 10.5935/0004-2749.2023-0054

**Published:** 2024-03-27

**Authors:** Anna Carolina Zamperlini Ferreira, Lucas Pitrez Mocelin, Fábio Zanini, Myrna Serapião dos Santos, Herberto José Chong-Neto, Márcia Carvalho Mallozi, Dirceu Solé

**Affiliations:** 1 Faculty of Medicine, Escola Paulista de Medicina, Universidade Federal de São Paulo, São Paulo, SP, Brazil; 2 Department of Collective Health, Faculty of Medicine, Universidade Federal do Pampa, Bagé, RS, Brazil; 3 Department of Ophthalmology, Escola Paulista de Medicina, Universidade Federal de São Paulo, São Paulo, SP, Brazil; 4 Cornea/Corneal Transplant Sector, Hospital de Olhos Paulista-HOLHOS, São Paulo, SP, Brazil; 5 Department of Pediatrics, Universidade Federal do Paraná, Curitiba, PR, Brazil; 6 Department of Pediatrics, Faculdade de Medicina do ABC, Santo André, SP, Brazil; 7 Outpatients Ambulatory, Division of Allergy, Clinical Immunology, Department of Pediatrics, Escola Paulista de Medicina, Universidade Federal de São Paulo, São Paulo, SP, Brazil; 8 Division of Allergy, Clinical Immunology and Rheumatology, Department of Pediatrics, Escola Paulista de Medicina, Universidade Federal de São Paulo, São Paulo, SP, Brazil

**Keywords:** Conjunctivitis, allergic, Keratoconjunctivitis, Hypersensitivity, Quality of life, Child, Surveys and questionnaires

## Abstract

**Purpose:**

The prevalence of ocular allergy varies according to the population and location of the study. Severe forms of ocular allergy are associated with compromised quality of life. In this study, we aimed to evaluate the application of the Brazilian-Portuguese version of the Quality of Life in Children with Keratoconjunctivitis questionnaire to children and adolescents with different subtypes of allergic conjunctivitis.

**Method:**

A total of 48 patients (aged 5-12 years) with allergic conjunctivitis were included in this study. They were enrolled and monitored at a specialized center. After the clinical appointment, the children responded to the questionnaire on two occasions at an interval of 30 days. Individual scores (ranging from 0 to 3) of the 16 items were added.

**Results:**

The Brazilian-Portuguese version of the Quality of Life in Children with Keratoconjunctivitis questionnaire demonstrated good translation, adaptation, and intellectual properties, with substantial internal consistency (Cronbach’s α coefficient = 0.702). There was no significant difference between the responses of the two interviews, revealing good reproducibility. The moderate/severe forms of allergic conjunctivitis had significantly higher quality of life scores (indicating a poorer quality of life) than the mild forms.

**Conclusions:**

The Brazilian-Portuguese version of the Quality of Life in Children with Keratoconjunctivitis proved to be quick, reliable, and reproducible for assessing the quality of life in children with allergic conjunctivitis. However, its ability to detect changes resulting from symptom aggravation or treatment needs to be further evaluated.

## INTRODUCTION

Allergic conjunctivitis (AC) is characterized by an immune-mediated inflammatory process on the eye’s anterior surface, usually in response to environmental allergens, such as mites, pollen, and animal dander^(1^,^2)^.

The prevalence of AC varies according to the population. In patients with allergic rhinitis, the prevalence of AC is reportedly 30%-71%^(3)^. In the general population, the prevalence of isolated AC is 6%-30%, with the seasonal form being the most common (40%) according to ophthalmological surveys^(3^,^4)^. In Brazil, a study among adolescents from Curitiba using a validated instrument revealed that the prevalence of AC (three or more episodes of eye itching in the previous year) was 20.7%, with a predominance among girls^(5)^.

The following ophthalmic conditions are included under the AC umbrella: seasonal AC (SAC) and perennial AC (PAC), which are associated with immediate hypersensitivity reactions; vernal keratoconjunctivitis (VKC) and atopic keratoconjunctivitis (AKC), which are more severe chronic forms and have an eosinophilic component; and papillary conjunctivitis, which is associated with a delayed hypersensitivity reaction^(2^,^4)^.

VKC and AKC have different clinical and pathophysiological characteristics and occur less frequently than SAC and PAC. They are potentially more severe and require ophthalmological follow-up to confirm the diagnosis, receive appropriate treatment, and avoid potential vision loss^(2^-^4^,^6)^. Furthermore, they compromise the patients’ and their caregivers’ quality of life (QoL)^(4^,^7)^.

Health-related QoL (HRQoL) is assessed based on the physical, psychological, and social components and can be affected by the individual’s perception of their disease and clinical conditions^(8^,^9)^. Individual experiences with the disease may influence the HRQoL more than its severity^(10)^.

Several instruments, including general^(11)^ and specific^(12^-^15)^, have been developed and are used to assess HRQoL in children with asthma and other allergic disea-ses. Among the instruments used for the assessment of the HRQoL of patients with ocular allergy, most assess patients with rhinoconjunctivitis^(16^-^21)^ and only a few assess patients with more severe conditions^(22^,^23)^.

In the present study, we evaluated the application of the Brazilian-Portuguese version of the Quality of Life in Children with Keratoconjunctivitis (QUICK) ques-tionnaire^(23)^ to patients with different types and severities of AC, except papillary conjunctivitis.

## METHODS

All patients diagnosed with severe AC who required ophthalmological evaluation and treatment between June 2019 and March 2020 were evaluated. In total, 48 patients (aged 5-12 years; male = 36) with AC (selected via convenience sampling) who were assessed by the Allergy, Clinical Immunology, and Rheumatology Unit of the Department of Pediatrics and the Department of Ophthalmology, *Escola Paulista de Medicina*, Federal University of São Paulo were included.

The diagnosis of AC was confirmed by an ophthalmologist and based on the presence of a triad of symptoms: conjunctival hyperemia, ocular pruritus and edema^(24)^, and/or tearing^(25)^. No patient had papillary conjunctivitis. According to the medications administered to the patients for the treatment of conjunctivitis, they were categorized as having mild, moderate, or severe disease. Patients with mild AC were those who were treated with ocular lubricants, antihistamine eye drops, topical nasal corticosteroids, and oral antihistamines^(25)^. Patients with moderate AC were those treated with ocular lubricants, corticosteroid eye drops, and oral corticosteroids or systemic immunosuppressants. Patients with severe AC were those treated with medications such as ocular lubricants, corticosteroid eye drops, and oral corticosteroids or topical/systemic immunosuppressants and/or had corneal lesions^(25)^.

Other variables such as age, sex, age at symptom onset, age at diagnosis, and personal and family history of allergic diseases were also evaluated.

### QoL questionnaire

During a scheduled medical appointment, patients answered the QUICK questionnaire consisting of 16 items divided into 2 domains: symptoms and limitations of daily activities. Initially developed in Italian, it was published in English after compliance with the rules for translation and back-translation of written questionnaires used in research^(23)^.

The English questionnaire was independently translated into Brazilian-Portuguese by two medical professionals with knowledge of the language and expertise in allergy. The translations obtained were compared by an expert committee, and no discrepancies were detected. Subsequently, the final version was independently back--translated by another translator whose native language was English, and it was compared to the original English version. Although there were a few discrepancies, the meaning was not compromised. Thus, we could use the Brazilian-Portuguese questionnaire (Supplementary files) in our study.

All evaluated items, referring to the last 2 weeks, were scored according to the frequency of occurrence (1 = never, 2 = sometimes, and 3 = always). The sum of all 16 items generated a total score of 16-48 points^(23)^.

After an average of 30 days after the first evaluation, the patients were re-evaluated and they answered the QUICK questionnaire again. On both occasions, the patients were directly observed by one of the investigators. For illiterate children, the investigator read out the questions under the supervision of a guardian.

The data obtained were recorded in a Microsoft Excel spreadsheet (2013 version; IBM Corp. Released 2013. IBM SPSS Statistics for Windows, Version 22.0. Armonk, NY: IBM Corp). Student’s t-test, paired t-test, Cronbach’s alpha coefficient, intraclass correlation coefficient (ICC), and Kappa concordance coefficient^(26)^ were used for analyzing the results; a rejection level of 5% was set as the null hypothesis.

The study was approved by the Ethics Committee of the Federal University of São Paulo - Hospital São Paulo. Informed consent was obtained from the parents or guardians of all patients. Microsoft Excel was used for the descriptive analysis of the data obtained.

## RESULTS

The clinical characteristics of the patients are listed in table 1. The patients were predominantly male, and there was a gap of at least 2 years between the onset of symptoms and the diagnosis of AC. All patients had a history of allergic rhinitis, 87.5% had asthma, and 79.1% had atopic dermatitis. Approximately 50% of the patients had a family history of allergic rhinitis or asthma, and 18.8% and 6.3% had a family history of atopic dermatitis and AC, respectively. Most of the patients had severe conjunctivitis, and >80% reported being under treatment (Table 1). The mean time required to complete the QUICK questionnaire was 7 minutes (range: 5-12 minutes).

**Table:** Quality of Life in Children with Vernal Keratoconjunctivitis questionnaire: Brazilian-Portuguese version.

Durante as duas últimas semanas, por causa da conjuntivite		
1 - ... você sentiu seus olhos queimarem?		
□ 1. Nunca	□ 2. Algumas vezes	□ 3. Sempre
2 - ... você teve problemas para ficar em ambiente com ar-condicionado?		
□ 1. Nunca	□ 2. Algumas vezes	□ 3. Sempre
3 - ... você teve que usar lenços?		
□ 1. Nunca	□ 2. Algumas vezes	□ 3. Sempre
4 - ... seus olhos incharam?		
□ 1. Nunca	□ 2. Algumas vezes	□ 3. Sempre
5 - ... você teve problemas em ambientes iluminados?		
□ 1. Nunca	□ 2. Algumas vezes	□ 3. Sempre
6 - ... você teve lacrimejamento ocular?		
□ 1. Nunca	□ 2. Algumas vezes	□ 3. Sempre
7 - ... você ficou com coceira nos olhos?		
□ 1. Nunca	□ 2. Algumas vezes	□ 3. Sempre
8 - ... você ficou com os olhos vermelhos?		
□ 1. Nunca	□ 2. Algumas vezes	□ 3. Sempre
9 - ... você ficou com a visão turva?		
□ 1. Nunca	□ 2. Algumas vezes	□ 3. Sempre
10 - ... você teve secreção ocular?		
□ 1. Nunca	□ 2. Algumas vezes	□ 3. Sempre
11 - ... você precisou usar colírios?		
□ 1. Nunca	□ 2. Algumas vezes	□ 3. Sempre
12 - ... você teve, pela manhã, olhos fechados e grudentos?		
□ 1. Nunca	□ 2. Algumas vezes	□ 3. Sempre
13 - ... você teve problemas para brincar ao ar livre?		
□ 1. Nunca	□ 2. Algumas vezes	□ 3. Sempre
14 - ... você teve problemas para praticar esportes (futebol, academia)?		
□ 1. Nunca	□ 2. Algumas vezes	□ 3. Sempre
15 - ... você teve dificuldades para fazer amizades?		
□ 1. Nunca	□ 2. Algumas vezes	□ 3. Sempre
16 - ... você teve problemas para ir a piscinas?		
□ 1. Nunca	□ 2. Algumas vezes	□ 3. Sempre

**Table:** Quality of Life in Children with Vernal Keratoconjunctivitis questionnaire: back-translated into English

During the last 2 weeks, because of conjunctivitis......		
1 - ... did you feel burning in your eyes?		
□ 1. Never	□ 2. Sometimes	□ 3. Always
2 - ... did you have trouble staying in air-condiotined room?		
□ 1. Never	□ 2. Sometimes	□ 3. Always
3 - ... did you have to use tissues?		
□ 1. Never	□ 2. Sometimes	□ 3. Always
4 - ... did you have puffy eyes?		
□ 1. Never	□ 2. Sometimes	□ 3. Always
5 - ... did you have problems in the light?		
□ 1. Never	□ 2. Sometimes	□ 3. Always
6 - ... did you have tearing?		
□ 1. Never	□ 2. Sometimes	□ 3. Always
7 - ... did you have itchy eyes?		
□ 1. Never	□ 2. Sometimes	□ 3. Always
8 - ... did you have red eyes?		
□ 1. Never	□ 2. Sometimes	□ 3. Always
9 - ... did you have blurred vision?		
□ 1. Never	□ 2. Sometimes	□ 3. Always
10 - ... did you have eye secretions?		
□ 1. Never	□ 2. Sometimes	□ 3. Always
11 - ... did you have to use eye drops?		
□ 1. Never	□ 2. Sometimes	□ 3. Always
12 - ... did you have closed and sticky eyes in the morning?		
□ 1. Never	□ 2. Sometimes	□ 3. Always
13 - ... did you have trouble playing outdoors?		
□ 1. Never	□ 2. Sometimes	□ 3. Always
14 - ... did you have trouble practicing sports (e.g., football and gym)?		
□ 1. Never	□ 2. Sometimes	□ 3. Always
15 - ... did you have trouble meeting your friends?		
□ 1. Never	□ 2. Sometimes	□ 3. Always
16 - ... did you have trouble going to swimming pool?		
□ 1. Never	□ 2. Sometimes	□ 3. Always

**Table 1:** Clinical characteristics of patients with allergic conjunctivitis (n=48) who were assessed using the Quality of Life in Children with Vernal Keratoconjunctivitis Questionnaire adapted for the Brazilian population

Characteristics	Values
Male (%)	75
Age (mean ± SD, years)	8.9 ± 2.3
Onset age (mean ± SD, years)	4.1 ± 2.5
Age at diagnosis (mean ± SD, years)	6.1 ± 2.3
Personal history	
Allergic rhinitis (%)	100
Asthma (%)	87.5
Atopic dermatitis (%)	79.1
Family history	
Allergic rhinitis (%)	50
Asthma (%)	52
Atopic dermatitis (%)	18.8
Allergic conjunctivitis (%)	6.3
Severity	
Mild (%)	16
Moderate (%)	12.5
Severe (%)	66
Treatment	
Allergic conjunctivitis (%)	81.3
Allergic rhinitis (%)	91.6

SD= standard deviation.

The reliability of the questionnaire was assessed via internal consistency by calculating the Cronbach’s α coefficient, which was 0.702. This indicated substantial consistency, with no differences in the symptoms and daily activities. Reproducibility was assessed using ICC.

Table 2 lists the average value of each item of the QUICK questionnaire at the time of enrollment and after 30 days. Most of the values were not significantly different, except for “burning eyes sensation”, “ocular swelling”, “ocular discharge” (symptoms), and “going to the pool” (physical activities). Based on the mean total score, there was no difference in the reproducibility, which proved to have excellent concordance (0.839).

**Table 2:** Mean scores and 95% confidence interval (95% CI) of the responses provided by children and adolescents with ocular allergy to the Quality of Life in Children with Vernal Keratoconjunctivitis questionnaire at the time of enrollment (Initial) and after approximately 30 days (Final)

Question	Initial			Final		
	Mean	95% CI	Mean		95% CI	Wilcoxon
Related symptoms						
Burning eyes	1.35	1.18-1.53	1.71		1.52-1.90	0.01
Air-conditioning	1.62	1.41-1.84	1.60		1.40-1.81	0.46
Use of tissue	1.83	1.63-0.04	1.85		1.64-2.07	0.42
Puffy eyes	2.04	1.86-2.22	1.88		1.70-2.05	0.02
Visual issues in the light	1.97	1.72-2.11	1.88		1.67-2.08	0.49
Tearing	2.04	1.83-2.25	2.19		2.05-2.33	0.06
Itchy eyes	2.48	2.33-2.63	2.46		2.30-2.61	0.06
Red eyes	2.29	2.11-2.47	2.17		1.99-2.34	0.13
Blurred vision	1.54	1.36-1.72	1.67		1.47-1.86	0.12
Eye secretions	1.67	1.50-1.84	1.90		1.70-2.10	0.01
Use of eye drops	2.38	2.18-2.57	2.33		2.11-2.56	0.35
Closed sticky eyes	1.88	1.72-2.03	1.75		1.60-1.90	0.09
Limitations of daily life						
Playing indoors	1.65	1.43-1.86	1.73		1.52-1.94	0.22
Practicing sports	1.33	1.17-1.50	1.46		1.26-1.66	0.13
Meeting friends	1.08	1.00-1.16	1.15		1.03-1.27	0.14
Swimming	1.71	1.46-2.00	1.98		1.72-2.24	0.05
Total score						
	28.80	27.55-30.08	29.69		28.14-31.23	0.21
Intraclass correlation coefficient = 0.839.						

**Figure 1:**
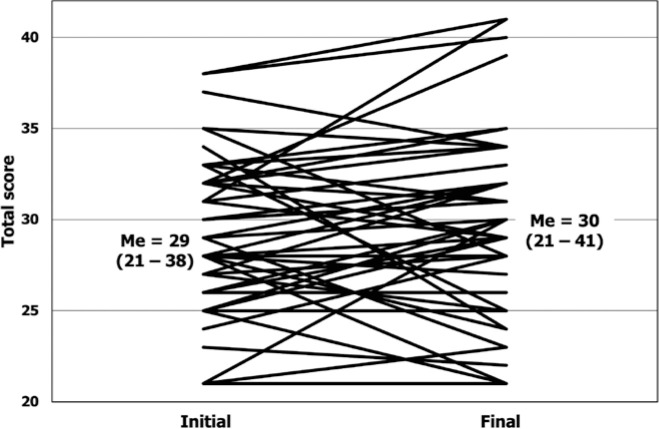
Total scores and median values (Me; variance of values) at the initial and final assessments. Each line represents a patient.

Figure 1 depicts the total QUICK score and the group median values and range at the time of enrollment and the last assessment.

Table 3 lists the frequency of patients’ responses accor-ding to the intensity (never, sometimes, and always) of the items at the two evaluation time points. There was a significant but low concordance in all variables between the two time points, except for “burning eyes sensation” and “having eyes shut and sticky in the morning”.

**Table 3:** Distribution of patients according to the response to each question of the Quality of Life in Children with Keratoconjunctivitis questionnaire during the initial and final assessments

During the last two weeks, because of Conjunctivitis	Initial			Final			Kappa^*^
	Never	Sometimes	Always	Never	Sometimes	Always	
Did you feel your eyes burning?	34	11	3	19	24	5	0.165
Did you have trouble staying in air-conditioned rooms?	25	16	7	25	17	6	**0.297**
Did you use tissues?	16	4	8	17	21	10	**0.337**
Did you have puffy eyes?	8	30	10	12	30	6	**0.385**
Did you have visual problems in the light?	13	26	9	15	24	9	**0.248**
Did you have tearing?	11	24	13	2	35	11	**0.224**
Did you have itchy eyes?	0	25	23	1	24	23	**0.469**
Did you have red eyes?	4	26	18	5	30	13	**0.206**
Did you have blurred vision?	25	20	3	21	22	5	**0.384**
Did you have eye secretion?	19	26	3	14	25	9	**0.259**
Did you have to use eye drops?	5	20	23	9	14	25	**0.316**
Did you have closed sticky eyes in the morning?	10	34	4	14	32	3	0.101
Did you have trouble playing outdoors?	24	17	7	21	19	8	**0.257**
Did you have trouble practicing sports?	34	12	2	31	12	5	**0.343**
Did you have trouble meeting your friends?	44	4	0	42	5	1	**0.229**
Did you have trouble going to swimming pool?	26	10	12	19	11	18	**0.580**

Italic and bold text indicates significant values.

* - Excellent: 0.81-1.0; Good: 0.61-0.80; Moderate: 0.41-0.60; Low: 0.21-0.40; Very low: 0.21-0.0.

Table 4 lists the comparison of the mean total scores based on the AC severity at the time of enrollment and at the last visit. There was a significant difference in the mean total score at the time of enrollment between those with mild disease and those with moderate/severe disease. This was not observed in the final evaluation scores.

**Table 4:** Mean values (and standard deviation) of the total score of the Quality of life in children with vernal keratoconjunctivitis (QUICK) questionnaire of patients with ocular allergy according to the disease severity at the time of enrollment and after 30 days

QUICK	Mild	Moderate/severe	p-value^*^
Initial	28.3 ± 3.9	31.0 ± 4.1	0.043
Final	29.9 ± 5.5	31.0 ± 4.2	0.227

* Paired t-test.

## DISCUSSION

In this study, we evaluated the Brazilian-Portuguese version of the QUICK questionnaire, which was designed to assess the HRQoL of 4-12-year-old patients with VKC. This questionnaire was first developed and validated in the Italian language^(23)^. The first version, consisting of 51 items, was qualitatively assessed, and 9 items were excluded as they were redundant, ambiguous, and difficult to understand^(23)^.

The subsequent version, consisting of 42 questions, was used in a pilot study of 30 patients with VKC. The patients independently answered the extent to which each item had compromised their lives in the preceding 2 weeks. The questions with the highest scores were selected for the new version of the questionnaire, which consisted of 30 questions. That new version was applied to another group of children (aged 5-12 years old) to evaluate their psychometric properties and was compared with the German version of a generic QoL self-assessment questionnaire for children (KINDL)^(27)^. The 16 items with the highest internal consistency and correlation with KINDL were selected for the final questionnaire. These 16 items were categorized into two domains: symptoms and daily activities^(23)^. Subsequently, the QUICK questionnaire was translated into English, following the standards established for translation and back-translation of questionnaires, as indicated in the original work^(23)^.

The content validity of QUICK in Brazilian-Portuguese was adequate as indicated by the lack of significant discrepancies between the translated and back-translated versions prepared by the experts participating in the study^(26^,^28)^. The internal consistency assessed using Cronbach’s α coefficient proved to be substantial (α=0.702) and was slightly lower than that observed in initial validation studies^(23^,^26)^.

The evaluation of reproducibility using two tests administered at an average interval of 30 days showed a significant index of concordance for all items, except for “burning eyes sensation”, “ocular swelling”, “ocular discharge” (symptoms), and “going to the pool” (physical activities). We noted a significant concordance revealed by an ICC greater than 0.8, characterizing the questionnaire as excellent when analyzing the total score. Reassessment after 30 days may be a limitation of the study, because the worsening or improvement of symptoms during the initial 30 days could easily change the responses, making it difficult to interpret the results. An interval of no more than 10 days between administering the questionnaire would be preferable. However, translating and validating this questionnaire into Portuguese enabled comparisons of its results to other studies conducted in different populations within the same field.

Although QUICK was created to assess VKC, we extended its use to other patients with different types and severities of AC in this study. Our patients were categorized according to the symptoms and medications used. This may have biased the clinical categorization of our patients. Because the patients remained under treatment during the study, patients categorized as having a mild condition could possibly have a controlled disease. However, this should not have interfered with our analysis because only 16% of the evaluated patients were categorized as having a mild disease. We found that patients with a mild disease had a significantly lower total QUICK score at the first assessment than those with moderate/severe disease, reinforcing its usefulness in patients with other forms of AC, in addition to those with VKC.

Among the available and established questionnaires for the assessment of the HRQoL of patients with ocular allergy, with or without allergic rhinitis, three target children and adolescents: Rhinoconjunctivitis Quality of Life Questionnaire (PRQLQ)^(22)^, Adolescent Rhinoconjunctivitis Quality of Life Questionnaire (AdolRQLQ)^(20)^, and QUICK^(23)^.

Our group has previously adapted and validated the AdolRQLQ questionnaire to assess adolescents with allergic rhinoconjunctivitis. The adapted questionnaire was highly reliable and proved to be reproducible and responsive, reinforcing its use during the follow-up of patients with allergic rhinoconjunctivitis^(29)^.

In a recent study, Mikhail et al. analyzed the established and commonly used tools for the assessment of the HRQoL of children, adolescents, and adults with allergic rhinoconjunctivitis and identified the advantages and disadvantages of each tool^(30)^. Compared with PRQLQ and AdolPRQLQ, QUICK has a faster response time and does not contain questions regarding emotional, school, and sleep issues^(30)^.

In conclusion, the Brazilian-Portuguese version of the QUICK questionnaire proved quick, reliable, and reproducible for the assessment of the QoL of children up to the age of 12 years suffering from AC. However, its ability to indicate changes resulting from symptom aggravation or treatment needs to be further evaluated.
